# A Case Report of Extra-pericardial Tamponade From a Low-Level Fall

**DOI:** 10.7759/cureus.73824

**Published:** 2024-11-16

**Authors:** Gillian Judge, Waslat Bakhshi, Fiona Sands, Christine Comer, Bryan Castle

**Affiliations:** 1 Emergency Medicine, Mater Misericordiae University Hospital, Dublin, IRL; 2 Trauma Inpatient Service, Mater Misericordiae University Hospital, Dublin, IRL; 3 Cardiothoracic Surgery, Mater Misericordiae University Hospital, Dublin, IRL

**Keywords:** andexanet alfa, chest wall injury, extra-pericardial tamponade, fall from less than 2 meters, major trauma in older adults, mediastinal hematoma, reversal of direct oral anti-coagulants, rib fractures, tamponade

## Abstract

A 61-year-old man in critical condition was admitted to the resuscitation room in the emergency department, presenting with chest pain and shortness of breath. His medical history included recent treatment with oral antibiotics for pneumonia, long-standing chronic obstructive pulmonary disease (COPD), a 40-pack-year smoking history, and a left popliteal artery embolus. He was also on chronic medications, including apixaban and aspirin. Initially, the patient did not recall or volunteer a recent history of repeated falls from standing height.

An electrocardiogram (ECG) showed sinus tachycardia. A chest radiograph raised suspicion of an increased left-sided effusion, and a subsequent computed tomography pulmonary arteriography (CTPA) confirmed displaced fractures of the left sixth and seventh ribs. A large, expanding mediastinal hematoma measuring 15 cm in depth was also identified. An urgent CT aortogram was performed, revealing ongoing contrast extravasation without major vessel injury. The hematoma exerted a mass effect on the adjacent right ventricle, although no significant pericardial effusion or fluid was detected.

The anticoagulant effect of apixaban was rapidly reversed following specialized hematological advice, using andexanet alpha. This intervention's potential risks and benefits were carefully considered, particularly regarding heparin unresponsiveness and the complications that might arise if bypass surgery became necessary. The patient then underwent an emergency sternotomy, during which a large anterior mediastinal hematoma was successfully evacuated without complications. He ultimately made a full recovery.

Falls from less than 2 meters in height are becoming an increasing public health concern at a population level. In older patients, there should be a lower threshold for considering cross-sectional imaging. Many patients in this demographic are on direct oral anticoagulants, so it is crucial to consider and discuss the reversal of these agents with relevant multidisciplinary teams. This case highlights the complexities of polypharmacy and the medical challenges posed by the reversal agent andexanet alpha. Expanding mediastinal hematomas causing obstructive shock are rare, with most literature describing posterior rather than anterior mediastinal hematomas, particularly in cases resulting from a simple fall.

## Introduction

We present a case of a traumatic anterior mediastinal hematoma resulting in extra-pericardial cardiac tamponade that required urgent surgical intervention. The injury occurred after a fall from standing height in a patient who was taking direct factor Xa inhibitor, apixaban. A literature review of the past 30 years was conducted, revealing cases of mediastinal hematomas with both iatrogenic causes, such as post-central venous catheter insertion or post-percutaneous intervention, and traumatic causes. Mediastinal hematomas can signpost the presence of an aortic injury; although an important cause, they are not the most common cause of mediastinal hematomas [1].

Aortic injuries are typically linked to high-energy mechanisms such as motor vehicle accidents and pedestrians struck by vehicles [2]. A diagnosis of a mediastinal hematoma should prompt a computed tomography angiography (CTA) to investigate further for aortic injury given that one study estimated an overall mortality (prehospital and in-hospital) of 59% [3]. The anterior mediastinal hematoma noted in this case was from an unidentified source in a patient with two rib fractures. The literature only describes three cases of mediastinal hematoma secondary to a fall from standing height, with all three being posterior hematomas and none requiring surgical intervention [4-6].

Falls from less than two meters are an increasing public health concern and are a leading cause of major trauma [7]. This case underscores the potential severity of injury that can result from a low-velocity mechanism of injury.

A considerable number of trauma patients present while on anticoagulant medications, posing a challenge to their management [8]. In recent years, the preferred anticoagulants have changed, making the reversal of various agents more complex [9]. With an increasing elderly population, these presentations and considerations will become more common, highlighting the importance of multidisciplinary discussion and decision-making.

## Case presentation

History

The patient is a 61-year-old male with a medical history that includes chronic obstructive pulmonary disease (COPD), a 40-pack-year smoking history, and a recent episode of pneumonia that was treated with oral antibiotics. His ongoing medical regimen involved chronic anticoagulation therapy with apixaban five milligrams twice daily (the appropriate dose for this 117 kg patient) and aspirin 75 mg daily due to a prior popliteal artery embolus. He presented to the Emergency Department (ED) with acute worsening of central to left-sided crushing chest pain that was pleuritic and exacerbated by movement. He reported four days of increasing shortness of breath on exertion, accompanied by intensifying pain. Additionally, he experienced two to three falls over the preceding four days, with the most recent fall occurring on the day of presentation.

Examination

Upon arrival at the ED, the patient’s vital signs included a heart rate of 105 beats per minute (bpm), blood pressure of 159/90 mmHg, respiratory rate of 24 breaths per minute, and oxygen saturation of 90% on room air. He was afebrile, with a temperature of 36.1 degrees Celsius, and his Glasgow coma scale (GCS) score was 15/15. Initial management included treatment for a suspected COPD exacerbation with three 2.5 mg salbutamol nebulizers and two 0.5 mg ipratropium nebulizers. However, due to a prolonged wait before being seen by a physician, his chest pain acutely worsened four hours after presentation. The patient was then transferred to the resuscitation room for further evaluation, but his repeat vital signs remained unchanged. A 12-lead electrocardiograph (ECG) showed a normal sinus rhythm at 98 bpm.

Laboratory results

Intravenous (IV) access was established, and blood samples were collected. Venous blood gas analysis was unremarkable (Table 1). The full blood count (FBC) showed a raised white cell count (WCC) and neutrophils of 12.72 x10^9/L and 10.95 x10^9/L. Creatinine, urea, and electrolytes were within normal limits. The C-reactive protein (CRP) and D-dimer were mildly elevated at 12 mg/L and 1.05 mg/L, and the troponin was normal.

**Table 1:** Venous blood gas, full blood count, C-reactive protein, D-dimer, and troponin results available after resuscitation room admission

	Lab Value	Normal Range
pH	7.4	(7.35-7.45)
pCO2	5.67kPa	(4.5-6.0kPa)
pO2	6.04kPa	(11-14.5kPa)
Lactate	1.4mmol/L	(0.5-2mmol/L)
Base Excess	1.2 mmol/L	(-2.3-2.3mmol/L)
White Cell Count	12.72 x10^9^/L	(3.5-11 x 10^9^/L)
Neutrophils	10.95 x10^9^/L	(2-8 x 10^9^/L)
Haemoglobin	13.8 g/dl	(13-18g/dl)
Platelets	301 x10^9^/L	(150-400x10^9^/L)
C- Reactive Protein	12 mg/L	(<7mg/L)
D-Dimer	1.05 mg/L	(0.00-0.50mg/L)
Troponin	5 ng/L	(<34ng/L)

Radiological imaging

**Figure 1:**
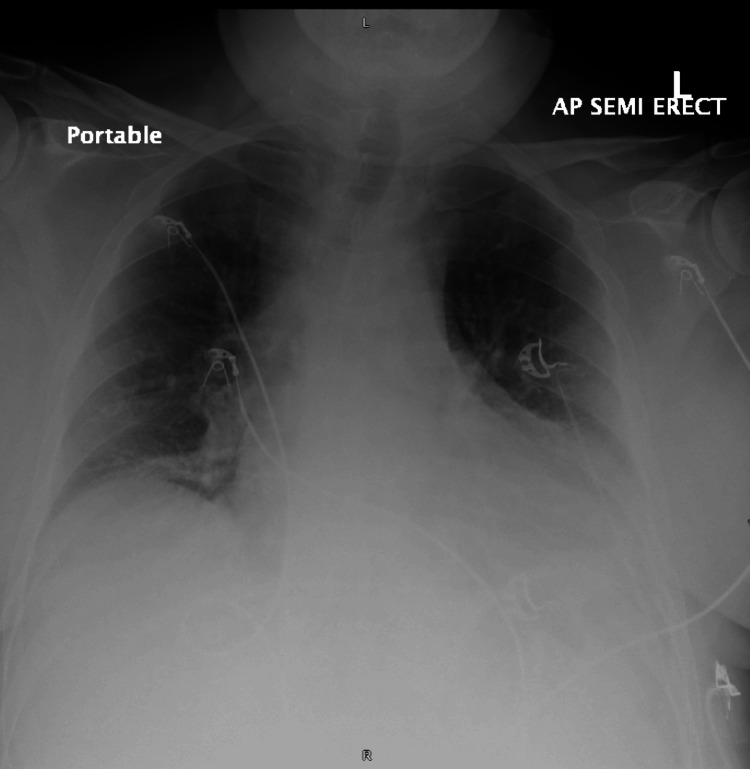
Portable chest X-ray in the resuscitation room

**Figure 2:**
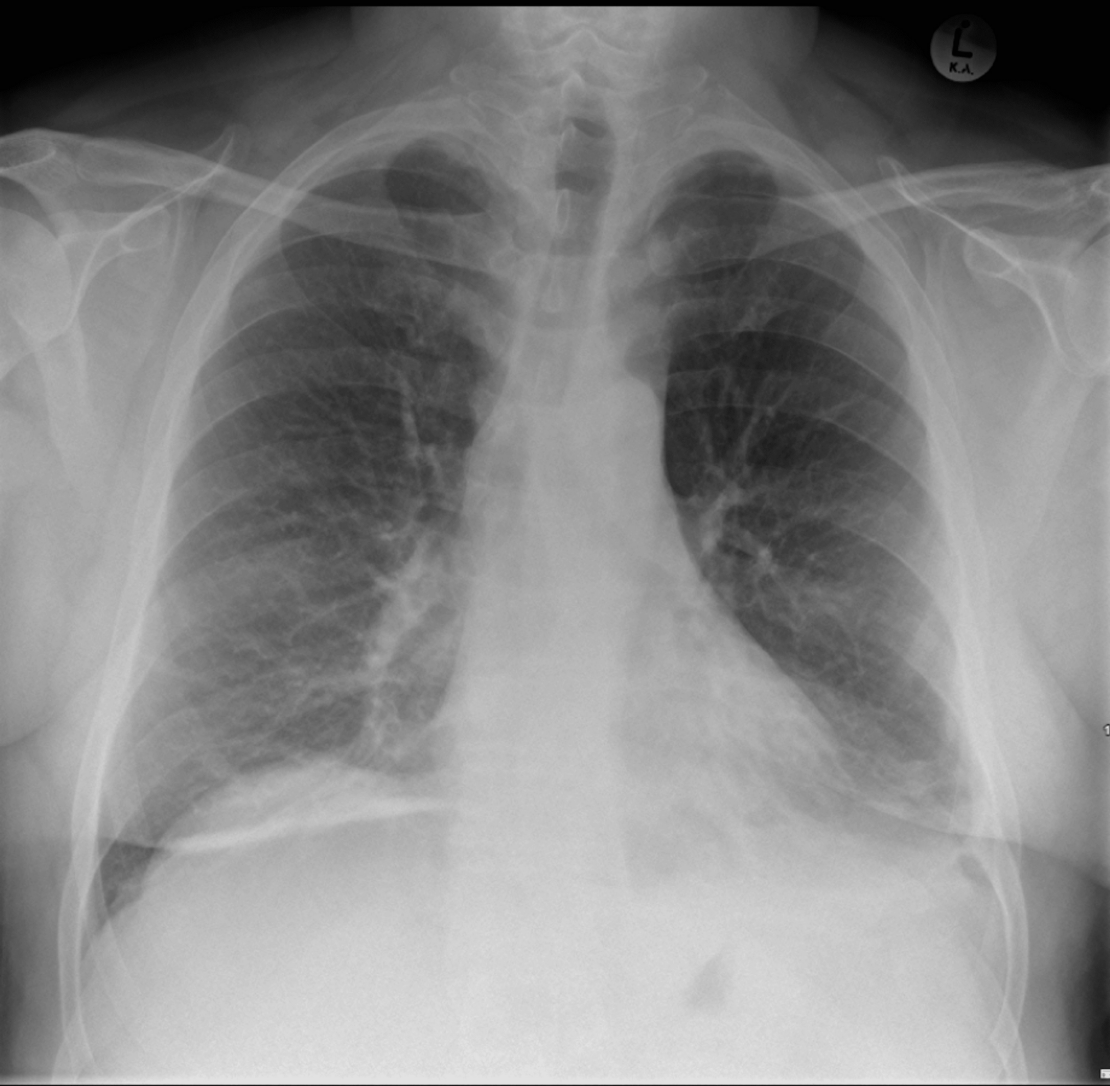
Chest X-ray from initial presentation with lower respiratory tract infection symptoms

**Figure 3:**
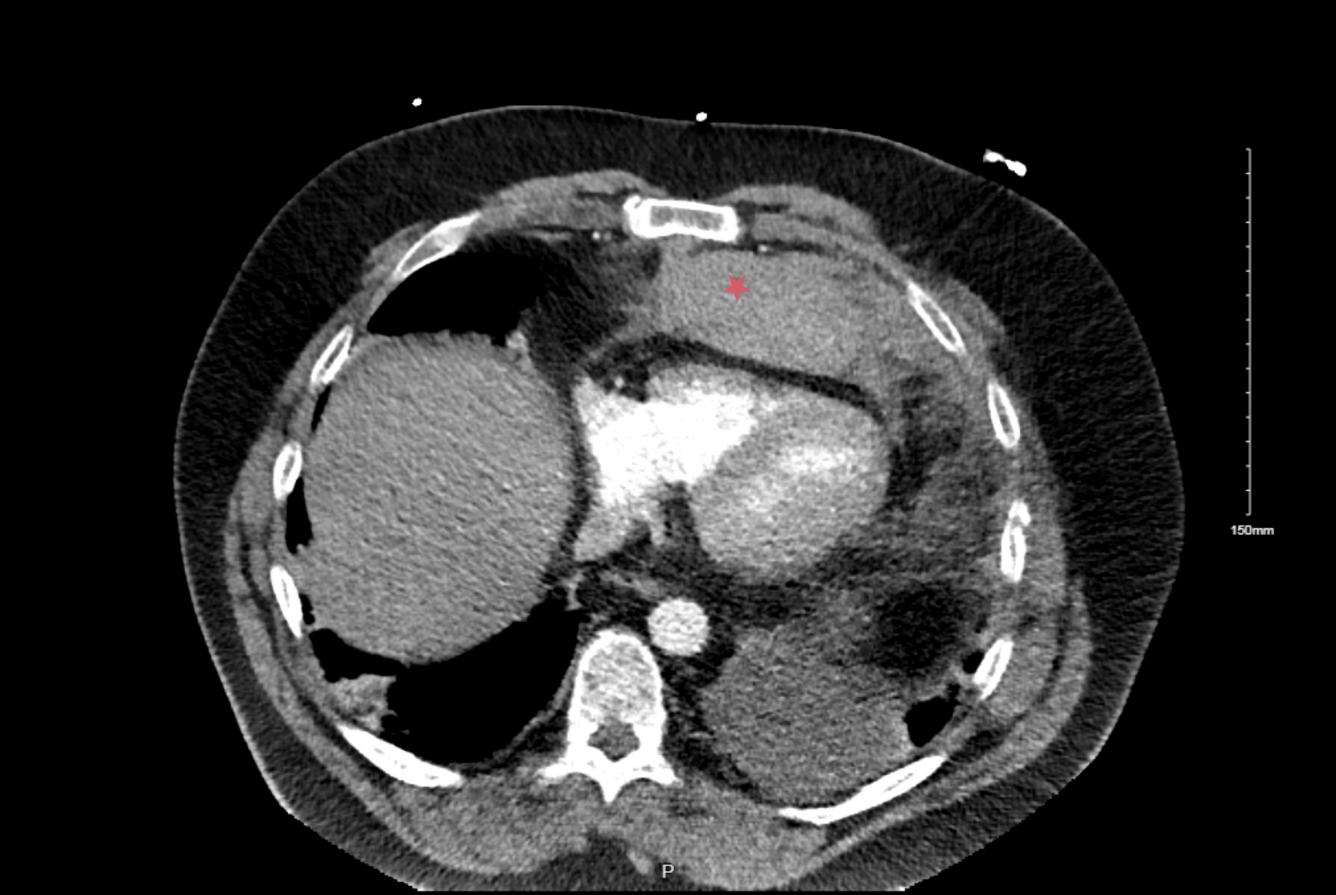
Axial view of the computed tomography pulmonary angiogram revealing a large anterior hematoma (red star)

**Figure 4:**
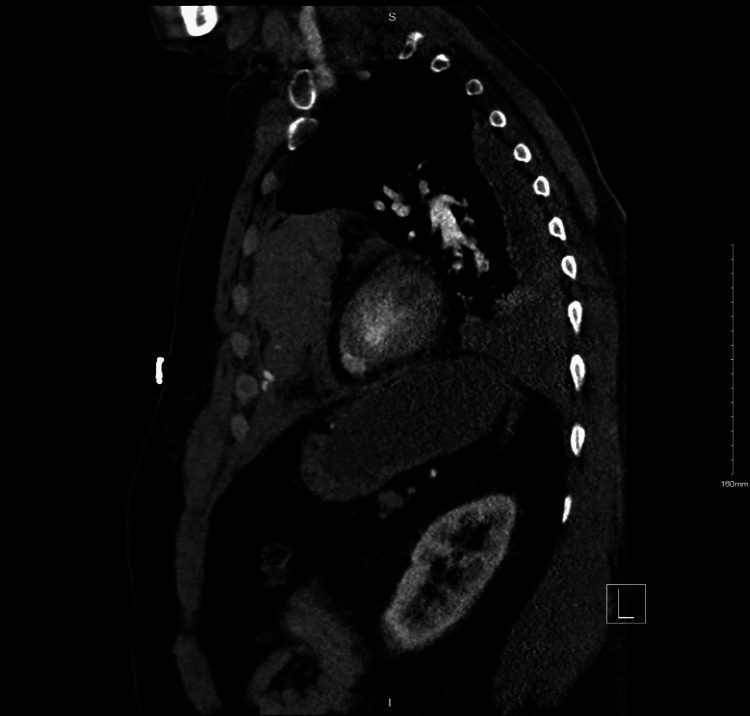
Sagittal view of the computed tomography angiography of the anterior mediastinal hematoma described

A portable chest X-ray demonstrated an increased size of a left-sided pleural effusion (Figure 1) compared to an X-ray taken four days earlier when the patient presented with symptoms of upper respiratory tract infection (Figure 2). There was evidence of bi-basal atelectasis and increased airspace opacification in the retrocardiac region, likely representing atelectasis. No obvious rib fractures were noted. Due to the worsening of his central chest pain and a history of syncopal episodes, a CTPA was performed. The CTPA ruled out pulmonary embolism but revealed mildly displaced fractures of the left sixth and seventh ribs and costal cartilage. A large anterior mediastinal hematoma measuring 15 cm x 12.5 cm x 5 cm was identified (Figure 3). A small area of increased density within the hematoma raised concern for active contrast extravasation, prompting a dedicated triple-phase CT scan (Figure 4). This scan confirmed active extravasation within the hematoma, which had increased in size and was exerting a mass effect on the right ventricle. The case was discussed with an interventional radiologist, but no intervention was pursued due to the absence of an identifiable bleeding vessel.

Management and outcome

Upon reassessment, the patient's condition deteriorated with increasing pain and signs of hemodynamic compromise. His heart rate increased to 110 bpm, and his blood pressure dropped to 60/40 mmHg. Given the ongoing anticoagulation therapy, prothrombin complex concentrate was administered, and the case was discussed with the on-call hematologist. It was decided to reverse the effects of apixaban using a low-loading dose of recombinant factor Xa andexanet alfa. The on-call cardiothoracic surgery team was consulted, and the decision was made to take the patient urgently to the operating room for evacuation of the hematoma. The patient underwent a median sternotomy (approximately two hours after andexanet alfa had been administered) during which clots were found in the anterior mediastinum and over the anterior pericardium, compressing the right ventricle. These clots were removed, along with additional clots in the pericardial fat, though the pericardium itself was not entered. The left pleura was opened, and clots and blood were evacuated. Rib fracture points were identified, with no active source of bleeding identified during the procedure. Three 28 French chest drains were inserted: two in the mediastinum and one in the left pleural space.

Postoperatively, the patient was admitted to the intensive care unit (ICU) for one night, extubated, and subsequently transferred to the cardiothoracic high-dependency unit, where he remained for three days. On the first postoperative day, therapeutic enoxaparin was withheld due to high drain output, which measured 750 ml. Enoxaparin at a dose of 80 mg twice daily was resumed on the second postoperative day, and the drains were sequentially removed, with the final drain being removed on the fourth postoperative day. By the eighth postoperative day, the patient was reviewed by the vascular team, who advised restarting apixaban at a dose of 5 mg twice daily. Given the multiple flights of stairs in his home, it was determined that he would benefit from further rehabilitation before returning home. He was subsequently discharged to a step-down facility to continue his recovery.

## Discussion

According to the World Health Organization (WHO), falls account for 684,000 deaths annually, making them a significant and growing public health issue [5]. Most of these fatalities occur among adults over the age of 60, with males being more likely to die following a fall. Reflecting these global statistics, the "2021 Irish Major Trauma Audit" reported that 62% of all major traumas (4,055 patients) resulted from falls of less than 2 meters [10]. The average age of these patients was 62, and 55% of the injuries occurred at home. Notably, 39% of injuries in patients under 64 were due to falls from less than 2 meters.

"The Major Trauma Audit Report focused on older adults", revealing that chest injuries occurred in 14% of cases for those under 65 and in 16% of cases for those over 65. Additionally, 16% of patients injured at home sustained chest injuries [11].

Although the patient in this case was 61 years old, he had multiple comorbidities and was considered frail. "The Trauma in the Older Person Audit" emphasizes that the rate of major trauma from falls at standing height increases significantly in patients over 70. This highlights the heightened vulnerability of older adults to serious injuries from relatively minor falls [11].

While chest injuries are relatively common, anterior mediastinal hematomas resulting from trauma are unusual. An anterior mediastinal hematoma causing extra-pericardial tamponade and requiring surgical intervention after a simple fall is particularly unusual [12].

A PubMed search of "Mediastinal Haematoma" and "Mediastinal Hematoma for the years 1984-2024" returned 96 and 601 results, respectively. Four case reports of mediastinal hematomas following falls were identified [4-6,13]. One case involved a fall from 30 feet [13], while the other three were falls from standing height or "simple falls" [4-6]. All three of the latter described posterior mediastinal hematomas [4-6], contrasting with the anterior hematoma in this case. None of these cases required surgical intervention.

In 2007, Lakshmi reported a case of a 76-year-old woman who fell against a sink, presenting to the emergency department with shoulder pain and a desaturation to 85% [4]. A CT scan revealed a large posterior hematoma. In 2015, Thamamongood et al. described a 77-year-old patient presenting with neck swelling after a fall from a standing height [5]. A CT scan showed a hypodense mass in the bilateral carotid space, retropharyngeal space, and posterior mediastinum, diagnosed as a posterior mediastinal hematoma secondary to hyperextension of the neck, which spontaneously resolved. In 2012, Josse et al. reported a patient who fell against a toilet and was diagnosed with a C7 transverse process fracture and a posterior hematoma compressing the trachea [6]. The patient was intubated due to concerns of an expanding hematoma and received octaplex and vitamin K, as they were on warfarin, but surgical intervention was not required.

There were no cases of anterior mediastinal hematomas resulting from falls from standing height, nor any describing extra-pericardial tamponade from such mechanisms. Extra-pericardial tamponade has been described in relation to other blunt trauma mechanisms. A review by Hajjar et al. acknowledged that localized anterior hematomas compressing the right ventricle and outflow tract are rare [12]. The paper noted that while assessment of the aorta and heart after blunt trauma is well-recognized, bleeding external to the pleural spaces may occur from injuries to the intercostal or internal mammary vessels.

In recent years, the preferred anticoagulants have shifted. An article in the Journal of the American Heart Association estimated that the rate of anticoagulation in atrial fibrillation patients increased from 56.3% to 64.7%, with direct oral anticoagulant (DOAC) use rising from 4.7% in 2010 to 47.9% in 2020 [9]. One study estimated that 7.9% of trauma patients were anticoagulated, with 35.23% of those on a DOAC [8]. A retrospective German study categorized patients based on their antithrombotic medications and matched them to a control group not on anticoagulants [14]. The study found that DOAC therapy was associated with an increased coagulopathy rate (37.5% vs. 5.2%), higher rates of PCC transfusion, and an increase in surgical procedures compared to controls. There was no difference however in overall in-hospital or early mortality.

Hemorrhage remains the leading cause of mortality in trauma patients, many of whom are coagulopathic upon arrival at the hospital. Access to reversal agents for DOACs is crucial. While DOACs are not independently associated with mortality [14], the American Association for the Surgery of Trauma (AAST) conducted a prospective observational multicentre study from 2015-2018 on the initial experience with DOAC reversal in trauma patients [15]. The study found that 12% of patients did not undergo anticoagulation reversal, though older and more severely injured patients were more likely to receive reversal. As the study commenced shortly after Food and Drug Administration (FDA) approval for the monoclonal antibody idarucizumab for reversal of DOAC dabigatran and andexanet alfa for reversal of rivaroxaban and apixaban, reversal rates may have increased as familiarity with these agents grew.

## Conclusions

In conclusion, the literature review did not identify any previous cases of anterior mediastinal hematomas resulting from low-level falls, nor any that required surgical intervention. This case adds to the existing data, highlighting the serious risks posed by seemingly minor trauma or low-velocity mechanisms in anticoagulated and frail patients.

This underscores the need for heightened clinical vigilance and suspicion regarding severe injuries from low-velocity trauma in this patient group. Timely diagnosis, interdisciplinary collaboration, and prompt surgical management are vital in complex trauma care. As the elderly population increases and direct oral anticoagulant use rises, these considerations will become increasingly critical in trauma management.
